# Iatrogenic Proximal Urethro–Rectal Perforation During Foley Catheter Insertion

**DOI:** 10.1155/cris/4784527

**Published:** 2025-01-08

**Authors:** Claude Tayar, Ali Alameh, Rawan Abdallah, Jamil Boufarah, Yehya Tlaiss, Hadi Farhat

**Affiliations:** ^1^Department of General Surgery, Clemenceau Medical Center, Beirut, Lebanon; ^2^Department of General Surgery, University of Balamand, Beirut, Lebanon

**Keywords:** Foley catheterization, lower anterior resection, rectal perforation, urethral stenosis, urethro–rectal fistula, urethroscopy

## Abstract

Iatrogenic urethral–rectal perforation represents a rare but severe complication arising from medical interventions, notably highlighted in the context of Foley catheter insertion. This case report outlines the presentation, diagnosis, management, and outcomes of a 71-year-old male patient who experienced iatrogenic rectal perforation during the routine insertion of a Foley catheter, against the backdrop of several predisposing factors, such as atrial fibrillation, valvular disease, benign prostatic hyperplasia, urethral stenosis, and colorectal cancer with liver metastasis. The inadvertent creation of a rectourethral fistula during the procedure led to an urgent multidisciplinary approach involving surgery and postoperative management, including fecal and urine diversion and antibiotic therapy. The case highlights the critical importance of meticulous technique and comprehensive preoperative patient assessment in minimizing the risk of such iatrogenic complications. It further discusses the management strategies for rectourethral fistulas, ranging from conservative approaches to surgical interventions, and emphasizes the role of fecal diversion, urine diversion, and the potential of robotic surgery in enhancing outcomes for complex cases. The report concludes by reflecting on the intricate balance between routine medical procedures and the potential for severe complications, highlighting the need for heightened awareness and skill in the prevention and management of iatrogenic rectal perforation.

## 1. Introduction

Iatrogenic rectal perforation is a rare, but potentially serious complication of some medical procedures. It can be caused by many factors, including instrument insertion, excessive force, or inadequate lubrication [1]. One such procedure associated with rectal perforation is the insertion of a Foley catheter. While this is a routine procedure, it can still result in severe injury if performed incorrectly. According to recorded data, only 1.5% of catheterization days experience genitourinary trauma [2]. Several contributing risk factors can result in this severe complication, such as fragile rectal tissues caused by previous injuries or radiation exposure [3]. Furthermore, the type of Foley catheter and the technique of insertion can lead to this complication [4]. Complications of urethral catheterization include catheter-associated urinary tract infection (UTI), urethral trauma, including creation of a false passage at the level of the prostate gland or bladder neck, and urethral strictures [5]. In this case report, we present the case of a patient who experienced iatrogenic rectal perforation during the insertion of a Foley catheter, and the subsequent management of this complication. We aim to highlight the importance of careful technique and patient selection in minimizing the risk of such complications.

## 2. Case Presentation

The case is of a 71-year-old male known to have atrial fibrillation, valvular disease, status postbiological valve replacement 4 years ago, on Xarelto 15 mg (rivaroxaban), hypertension, benign prostatic hyperplasia, urethral stenosis, irritable bowel syndrome, and a history of hemorrhoids, presenting for a few months' history of abdominal cramps. The cramps were diffuse, associated with rectorrhagia and difficulty, to inability, to pass stools. He described his stool as “pencil-like.”

Colonoscopy showed a rectosigmoid mass around 5 cm from the anal verge extending up 7 cm. Biopsy taken showed moderately differentiated adenocarcinoma. A computed tomography (CT) scan showed the mass extending from midrectum up to the high rectum, significantly narrowing the lumen, in addition to a few bilobar liver lesions of different sizes, most likely metastases. Carcinoembryonic antigen (CEA) level was 524 ng/mL. The diagnosis of colorectal cancer with liver metastasis was made. Decision to do LAR was taken since it was obstructive, before initiating chemotherapy. Pathology report revealed invasive adenocarcinoma of stage pT4a, pN1, grade 2 moderately differentiated.

Intraoperatively, under general anesthesia, and after multiple failed trials, the insertion of a Foley catheter was performed. After the LAR was done and while performing the colorectal anastomosis, during the digital rectal exam (DRE) and before the insertion of the end-to-end anastomosis (EEA) stapler, the Foley catheter tip balloon was found in the rectum. Urgent urology consult was requested where an intraoperative urethroscopy was performed that showed a false tract in the urethra with a connection between the membranous urethra and the rectum. Silicone Foley was inserted under guidance and was advised to be kept 3 weeks postoperative. A 28 mm EEA stapler was used to perform an end-to-end stapled anastomosis. Donuts were inspected and found to be intact.

Alas, the decision was taken, after discussion with the urologist, that a diverting ileostomy will be a good option for protection and to exclude the fistula tract giving it time to heal. A diverting ileostomy was performed and planned to be reversed 6–8 weeks postoperative.

Postoperative, the patient was clinically and hemodynamically stable and was placed on metronidazole and ciprofloxacin combination therapy, the course of which he would finish at home after discharge, for a total of 14 days keeping in mind the fistula and the location operated on. The patient was giving good clear urine output, physical exam was unremarkable with a soft abdomen, tender in the suprapubic region and around the trocar's insertion site.

On day 8 postoperative, the patient's drain was removed and was set to follow-up with the urologist on day 21 postoperative to assess whether or not to remove the Foley catheter. After urography imaging that showed no leak and no urethero–rectal fistula, the Foley catheter was removed.

Eight weeks postoperative, CT scan of the abdomen and pelvis with intravenous, oral, and rectal contrast was done. Liver lesions were found as previously noted, in both lobes, and of different sizes. Ileostomy was noted, with no bowel obstruction. There was no evidence of leak or obstruction as the rectal contrast filled the colon and terminal ileum. The rectal anastomosis was unremarkable. The next day, reversal of the ileostomy was performed. The patient was clinically and hemodynamically stable and was discharged on day 2 postileostomy closure. He was then referred to an oncologist for chemotherapy sessions and management of his liver metastases.

## 3. Discussion

Rectourethral fistula is an uncommon complication of surgical interventions that lead to a significant reduction in quality of life. It may result from surgical injury, such as low anterior resection (LAR), as in our case, prostatectomy, abdominoperineal resection (APR), ablative therapies, and transurethral resection of the prostate (TURP). Most common causes of rectourethral fistulas are iatrogenic and related to the oncologist treatment of prostate cancers and occurring in 0.1%–3.0% of prostate cancer treatments. These are called simple fistulas and are less than 1.5 cm, resulting from surgical etiology. Complex iatrogenic rectourethral fistulas result from nonsurgical causes including brachytherapy, external beam radiotherapy, and ablative therapy, all of which include high-intensity focused ultrasound (HIFU).

The cause of the rectourethral fistula dictates how to manage it. Simple fistulas have high chances of spontaneous resolution or successful repair with appropriate fecal diversion (ileostomy, colostomy) and urine diversion through keeping a Foley catheter inserted [6]. Complex fistulas have a better prognosis if surgically repaired [6].

To identify the location of the fistula, and its relation to the sphincter, retrograde urethrogram and cystogram are used. The rectum can also be filled with contrast through the fistula allowing its visualization in relation to the anal sphincter.

In the case of complex fistulas, recurrent UTI, perineal sepsis, or abscesses, fecal diversion before reconstruction is recommended. In our case, we opted for fecal diversion through ileostomy to prevent any UTIs from occurring. In cases where surgery is required, robotic surgery has proved to have better results for reconstruction, as it allows for better visualization and exposure [7].

Finally, Dellimore, Helyer, and Franklin [8] recommend prospectively in such cases, to modify catheter production and create catheters featuring a hydrophilic, antibacterial, low-friction, and resilient coating. Additionally, they propose designing catheters with a less rigid tip, a flexible shaft, and a configuration that minimizes contact between the catheter and the urethra [8].

## 4. Conclusion

In this case, the patient had several underlying medical conditions that may have increased his risk of rectal perforation, including a history of hemorrhoids and irritable bowel syndrome, but most importantly, a history of urethral stenosis that he had not revealed preoperative. Additionally, the patient had a false tract in the urethra that was identified during intraoperative urethroscopy. This may have made the urethra more susceptible to injury during catheterization. Rectal perforation during Foley catheterization is a rare but potentially serious complication that can lead to significant morbidity and mortality if not managed appropriately.

## Data Availability

The underlying data supporting the results of our study are available upon request from the corresponding author Yehya Tlaiss at yehyatlaiss@hotmail.com. We aim to ensure the transparency and reproducibility of our research findings, and we are committed to providing access to the data underlying our results to facilitate further exploration and validation by the scientific community.
